# Is Maternal Serum Homocysteine a Novel Diagnostic Biomarker for Predicting Placenta-Mediated Disorders?

**DOI:** 10.7759/cureus.33768

**Published:** 2023-01-14

**Authors:** Pooja Ramesh, Sudha Sumathy

**Affiliations:** 1 Department of Obstetrics and Gynaecology, Amrita Institute of Medical Sciences, Kochi, IND

**Keywords:** pregnancy-induced hypertension, fetal growth restriction, placental insufficiency, homocysteine, biomarkers

## Abstract

Background

Uteroplacental insufficiency and related disorders, though a significant cause of undesirable maternal and fetal outcomes, are complex and poorly understood. The newer screening modalities are expensive and difficult to procure for day-to-day use in developing countries. This study aimed to examine the association of mid-trimester maternal serum homocysteine levels with maternal and neonatal outcomes.

Methodology

This was a prospective cohort study involving 100 participants between 18 and 28 weeks of gestation. The study was conducted at a tertiary care center in south India from July 2019 to September 2020. Maternal blood samples were analyzed for serum homocysteine levels and correlated with the third-trimester pregnancy outcomes. Statistical analysis was done, and diagnostic measures were computed.

Results

The mean age was found to be 26.8 ± 4.8 years. Of the participants, 15% (n = 15) were diagnosed with hypertensive disorders during pregnancy, while 7% (n = 7) had fetal growth restriction (FGR) and 7% (n = 7) were complicated by preterm birth. An elevated maternal serum homocysteine level was positively associated with adverse pregnancy outcome measures such as hypertensive disorders (p = 0.001), with sensitivity and specificity of 27% and 99%, respectively, and FGR (p = 0.03) with sensitivity and specificity of 28.6% and 98.6%, respectively. Moreover, a statistically significant outcome was noted with preterm birth before 37 weeks (p = 0.001) and a low Apgar score (p = 0.02). No association was established with spontaneous preterm labor (p = 1.00), neonatal birth weight (p = 0.42), and special care unit admission (p = 1.00).

Conclusions

Such a simple and affordable investigation has the potential to go a long way in the early diagnosis and management of placenta-mediated disorders in pregnancy during the antenatal period, especially in low-resource settings.

## Introduction

Uteroplacental insufficiency has proven to be a significant cause of undesirable maternal and fetal outcomes. The pathogenesis, diagnosis, and management of placenta-mediated disorders are incredibly complex and poorly understood. These disorders mainly include hypertensive disorders of pregnancy, such as gestational hypertension, pre-eclampsia, and eclampsia in the mother, and are often associated with adverse fetal outcomes, such as low birth weight, fetal growth restriction (FGR), and preterm birth complications [1].

Following the landmark paper by Robert et al., pre-eclampsia has become the center of extensive research. Still, sadly, only very few investigations in the antenatal period are reliable enough to be used in practice. One of those investigations involves using different serum biochemical markers such as pregnancy-associated plasma protein A (PAPP-A), activin, soluble FMS-like tyrosine kinase 1 (SFLT-2), placental growth factor (PlGF), inhibin A, and homocysteine, which have proven correlations with unfavorable pregnancy outcomes. Homocysteine, one of the common amino acids circulating in the bloodstream, is a thiol-containing amino acid formed from the intracellular demethylation of methionine through a multi-step process. A high level of circulating homocysteine in the blood, also known as hyperhomocysteinemia, makes one more prone to endothelial injury and further inflammation in the blood vessels. Recent studies have shown that elevated plasma homocysteine levels boost the risk of macrovascular disease and atherothrombosis. In a normal pregnancy, besides nutritional and lifestyle factors, homocysteine is also influenced by certain physiological factors, such as hemodilution, increased glomerular filtration rate, endocrinological changes, and increased demethylation of homocysteine or higher utilization of homocysteine by the fetus. These factors can be responsible for the considerable reduction under normal circumstances in homocysteine by mid-pregnancy [2-4].

Some studies in obstetrics have previously established that elevated serum homocysteine levels have a well-established association with placental disorders, including hypertensive disorders of pregnancy, placental abruption, placental infarction, FGR, recurrent pregnancy losses, neural tube defects, orofacial clefts, club foot, and Down syndrome. A few of these can be linked to certain factors that cause elevated homocysteine levels, including genetic defects and polymorphism (MTHFR genotype), deficiency of folic acid and vitamin B, hypothyroidism, certain medications, and chronic renal impairment [5]. However, they have also shown conflicting results concerning the relationship between placental insufficiency secondary to an aberration in the placental microvasculature [6].

Hypertensive disorders are one of the significant contributors to maternal mortality as per the most recent maternal mortality report by the World Health Organization, thereby making it crucial that these complications are detected early on to manage them promptly. It is widely believed that the failure of trophoblastic invasion and the consequent placental under-perfusion leads to the release of highly active inflammatory mediators and hormones into the maternal circulation, which is the primary mechanism of pre-eclampsia. Based on this theory, markers to assess the microvascular status of placental circulation helps in narrowing the focus toward the pregnancies at higher risk of adverse outcomes and, hopefully, increase the early detection rate of such disorders [7].

The hunt for the single most reliable screening test to diagnose this pathology has been on for many years. However, this may never even become a reality due to the complexity of this disorder which is only beginning to be explored. The newer screening modalities are expensive and difficult to procure for day-to-day use in developing countries. This study was targeted to examine the association of mid-trimester maternal serum homocysteine levels with maternal and neonatal outcomes.

## Materials and methods

This prospective cohort study involved 100 participants attending the antenatal clinic of Obstetrics and Gynaecology at Amrita Institute of Medical Sciences, Kochi, south India, from July 2019 to September 2020. The participants were randomly selected and included pregnant women between the gestational age of 18 and 28 weeks, aged between 20 and 40 years, with an uncomplicated singleton gestation from a spontaneous conception. Pregnancies complicated by pre-existing maternal comorbidities or previous pregnancies with fetal/chromosomal malformations were excluded. All participants recruited in the study were on folic acid supplementation as per the recommendations of the National Institute of Health and Family Welfare (NIHFW) under the Government of India.

The Research Division of the institution registered the study after approval from the Ethics Committee (IEC-AIMS-2017-OBS.GYNEC-377). Because there is no previously established standard nomogram for the total homocysteine (tHcy) levels in the second trimester of pregnancy, for the purpose of this study, the 95th centile of total serum homocysteine values was taken as the cut-off value (tHcy ≥9.7 mmol/L) based on the earlier study by Onalan et al. The sensitivity for predicting the outcome was 61.3% with a 95% confidence interval and 10% allowable error, giving a sample size of 100 [7].

Demographic and medical information of the participants were entered prospectively during the antenatal visits at the clinics. The date of the last menstrual period was used to assign the gestational age, which was confirmed by the early pregnancy scan at 11-13 weeks or a second-trimester scan in case the former was unavailable. Informed consent was obtained from all participants and 2 mL of blood was drawn from the participants under aseptic precautions for serum tHcy estimation. The patients were followed up until their delivery, and the main placenta-mediated outcome measures in the third trimester, such as maternal hypertension, FGR, and preterm birth, were examined. Neonatal outcomes such as the birth weight at delivery, Apgar score, and neonatal special care unit admissions were recorded. This information was obtained from the hospital’s electronic medical records and telephonic follow-up. A correlation was then established between mid-trimester maternal serum homocysteine levels with pregnancy outcomes.

Definition of outcome measures

Gestational hypertension was defined as blood pressure more than 140/90 mmHg after 20 weeks of gestation on two occasions four hours apart. Pre-eclampsia was defined as gestational hypertension with significant proteinuria. Birth weight of <2.5 kg was considered low birth weight. Estimated fetal weight less than the 10th percentile with abnormal Doppler changes (Barcelona protocol) was considered FGR.

Serum homocysteine concentration

Venous blood specimens were drawn under aseptic precautions from the antecubital vein and collected in ethylenediaminetetraacetic acid vacutainer blood-collecting tubes according to standard hospital venepuncture and sample collection guidelines. The blood samples were then placed on ice, and all specimens were immediately transported to the laboratory in less than 20 minutes. After centrifugation of the sample, the Roche/Hitachi Cobas c-systems were used to determine tHcy quantitatively.

Homocysteine enzymatic assay is based on a novel enzyme cycling assay that assesses the co-substrate conversion product instead of assessing co-substrate or homocysteine conversion products of homocysteine. In this assay, the oxidized homocysteine form is first reduced to the free form. This then reacts with a co-substrate, S-adenosylmethionine (SAM), to form methionine (Met) and S-adenosylhomocysteine (SAH), catalyzed by homocysteine S-methyltransferase (SAH) and assessed by coupled enzyme reactions where SAH is hydrolyzed into adenosine (Ado) and homocysteine by SAH hydrolase. Homocysteine is then cycled into the V conversion reaction to form a reaction cycle that amplifies the detection signal. The formed Ado is immediately hydrolyzed into inosine and ammonia. The last step is where the enzyme glutamate dehydrogenase (GLDH) catalyzes the reaction of ammonia with 2-oxoglutarate and NADH to form NAD+. The concentration of homocysteine in the sample is directly proportional to the amount of NADH converted to NAD+. The main reagents used were NADH, enzyme, and start reagents [8].

Statistical analysis

SPSS version 20.0 software (IBM Corp., Armonk, NY, USA) was used for statistical analysis. Categorical variables are presented using frequency and percentage. Continuous variables are presented using mean and standard deviation. Statistical significance regarding the association of serum homocysteine with maternal and neonatal outcome measures was tested using the chi-square test. A p-value of less than 0.05 was evaluated to determine the statistical significance of the outcome measures studied. Diagnostic measures such as sensitivity, specificity, positive predictive value (PPV), and negative predictive value (NPV) were also determined.

## Results

The study involved 100 participants. The mean age was 26.8 ± 4.8 years, and 38% (n = 38) were primigravidae. It was noted that 15% (n = 15) were diagnosed with hypertensive disorders during pregnancy, while 7% (n = 6) had FGR and 7% (n = 7) were complicated by preterm birth. The cut-off value for maternal serum homocysteine level (tHcy) was taken to be an arbitrary value of ≥9.7 mmol/L, which was the 95th centile, based on the study by Onalan et al. [7]. A p-value of less than 0.05 was considered to determine the statistical significance of the outcome measures studied.

**Figure 1:**
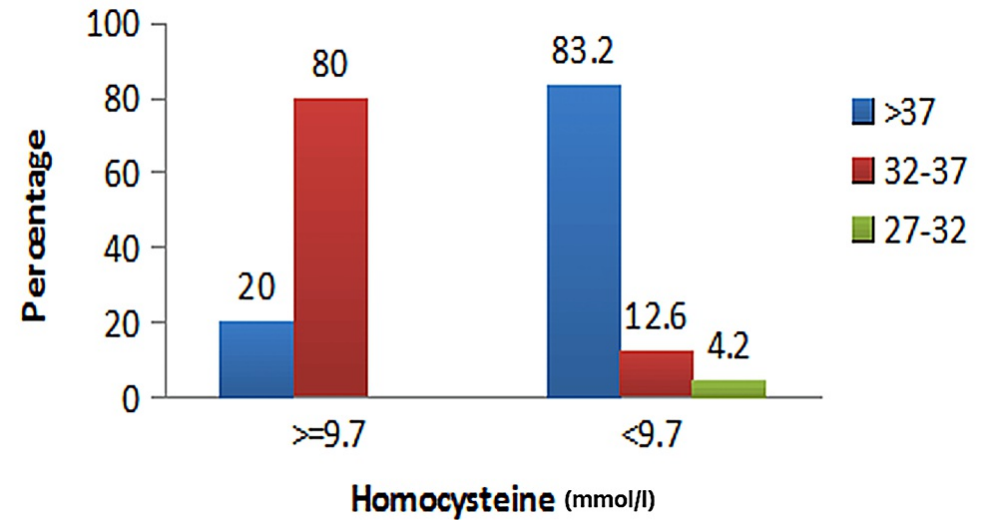
Graphical representation of serum homocysteine (total homocysteine) with gestational age at delivery.

**Figure 2:**
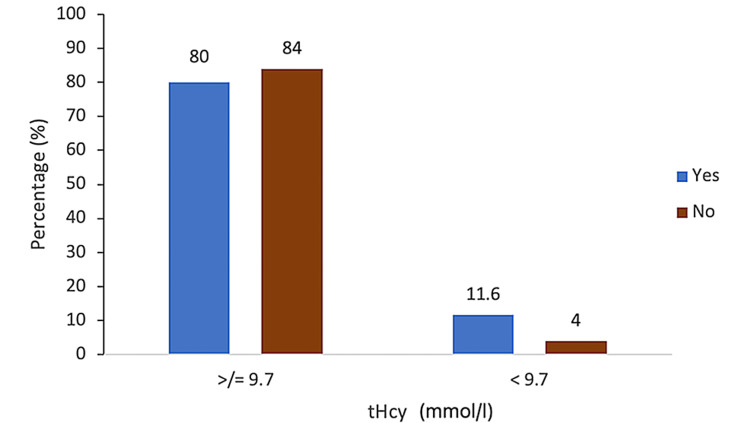
Graphical representation of serum homocysteine (tHcy) with hypertensive disorders. tHcy = total homocysteine

**Figure 3:**
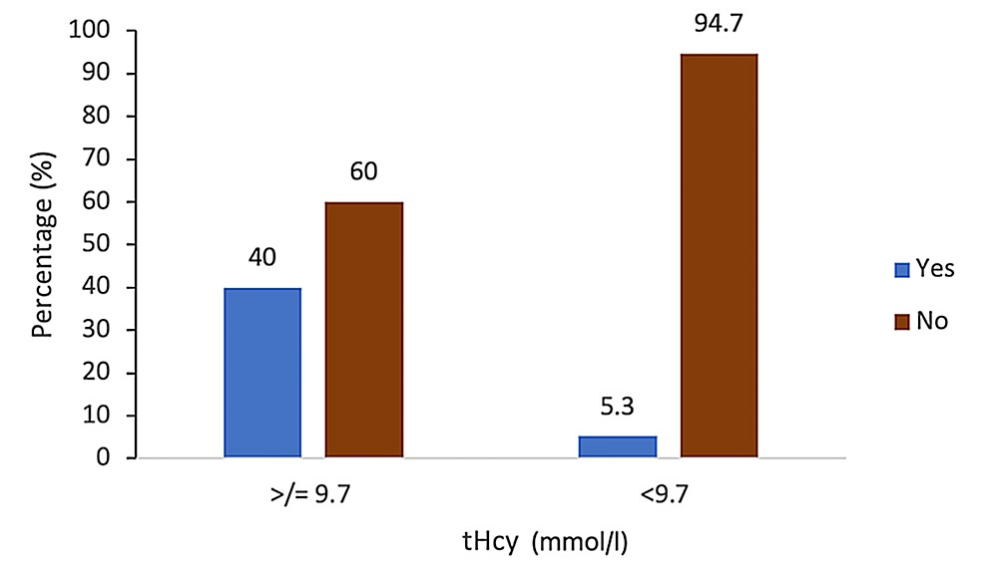
Graphical representation of serum homocysteine (tHcy) with fetal growth restriction. tHcy = total homocysteine

Of the 5% (n = 5) of the pregnancies with elevated tHcy, 80% (n = 4) delivered preterm before 37 weeks of gestation, with a statistically significant association (p = 0.001) (Figure 1). Statistical significance was also found with hypertensive disorders in pregnancy that contributed to 80% (n = 4) of the pregnancies with raised tHcy (p = 0.001), with a sensitivity of 27%, specificity of 99%, PPV of 80%, and NPV of 88.4% (Figure 2). Of the pregnancies with raised tHcy, 40% (n = 2) were linked to FGR (p = 0.039), with a sensitivity of 28.6%, specificity of 98.6%, PPV of 80%, and NPV of 94.7% (Figure 3). No association was found between elevated tHcy and spontaneous preterm labor. Furthermore, elevated homocysteine levels were not particularly related to the maternal body mass index or gestational diabetes mellitus and fetal anomalies in the present pregnancy.

**Figure 4:**
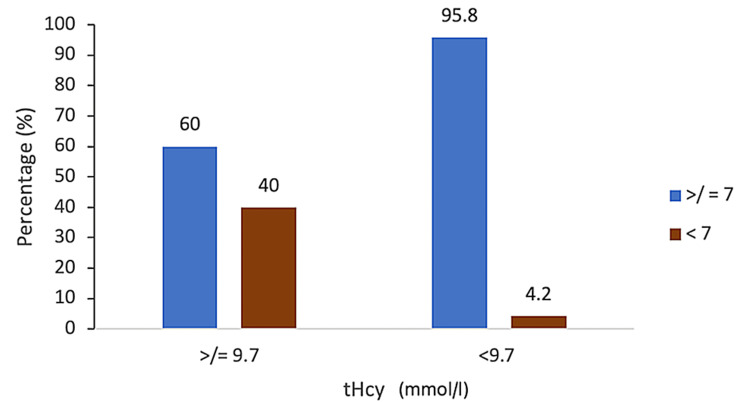
Graphical representation of serum homocysteine (tHcy) with Apgar score at birth. tHcy = total homocysteine

With regard to the neonatal outcome, 47% (n = 47) of the babies were born by cesarean section. An Apgar score of <7 was recorded in 6% (n = 6) of the 100 neonates born to the participants, of whom 33.3% (n = 2) were born to mothers with raised tHcy (p = 0.02), with a sensitivity of 3.2%, specificity of 67%, PPV of 60%, and NPV of 4.2% (Figure 4). Of the total neonates delivered, 8% (n = 8) required immediate neonatal intensive care unit admission for various reasons (p = 1). Of the pregnancies with raised tHcy, 40% (n = 2) of the neonates had a birth weight of less than 2.5 kg (p = 0.42). No significant association was noted between these measures and raised homocysteine.

## Discussion

Homocysteine, an amino acid, is a biomarker that has popularly emerged in clinical testing for various conditions over the last few decades. It is an intermediate metabolite that has a definite link with folate metabolism. Elevated homocysteine levels have been shown to have a detrimental effect on the human vascular endothelium. Although ample research has been conducted in the past involving the correlation between homocysteine and placental insufficiency, the results have been variable and inconsistent. It has been proven that homocysteine levels are elevated in placental vascular disease associated with maternal hypertensive disorders and intrauterine growth restriction. Rajkovic and colleagues published one of the earliest studies that considered the possible association between elevated homocysteine levels and pre-eclampsia, where they examined the levels of plasma tHcy irrespective of whether they had pre-eclampsia or not, and it was concluded that measurement of serum homocysteine concentration during pregnancy helps predict adverse pregnancy outcomes such as pre-eclampsia [9].

Again, consistent with the present study, a similar positive correlation was found by Maru et al., who studied the association of maternal serum homocysteine with hypertensive disorders, neonatal intensive care unit admissions, placental abruption, and multiorgan dysfunction. In this two-year study, they found that homocysteine levels were elevated in 93.3% of patients diagnosed with eclampsia and 86.67% of those diagnosed with maternal hypertension of varying severity. On analysis of the perinatal outcome, they found that 38 neonates born to mothers with hyperhomocysteinemia required admission to the neonatal intensive care unit. There were 20 stillbirths in the severe pre-eclampsia group compared to only two in the mild pre-eclampsia group [10].

Cotter et al. performed a case-control study with adequately matched controls for every case of non-severe pre-eclampsia. A vital query that needs to be addressed is whether serial testing of homocysteine is superior to a single measurement. Their study involved serial measurements until delivery and found a positive correlation with pre-eclampsia [11]. Laskowska et al. reported that normotensive patients with an elevated serum homocysteine level were associated with isolated intrauterine FGR. Homocysteine levels in women with pre-eclampsia were 1.8 times higher than their healthy counterparts at the time of diagnosis and immediately postpartum [12].

Powers and colleagues wanted to confirm whether the rise in homocysteine was specific to pre-eclampsia and verify the direct relationship between serum homocysteine levels and the degree of endothelial dysfunction. They concluded that vascular dysfunction of pre-eclampsia was not related to the magnitude of increased homocysteine concentration in the body and that there could be other unidentified intermediary factors that were likely to mediate that dysfunction [13].

Contrary to this study, in an Italian cohort study by D'Anna et al., 1,874 pregnant women who participated in a Down’s screening program were followed longitudinally for two years (2000 to 2001). Plasma homocysteine was measured in the mid-trimester of pregnancy and at delivery. Interestingly, the authors concluded that serum homocysteine was not relevant as an early predictor of pregnancy-related hypertensive disorders. Moreover, there was no difference between patients with isolated FGR and the controls [14]. There were also two other studies done in 2001, one by Hietala et al. and the other by Hogg et al., with both suggesting no association between elevated maternal homocysteine and hypertensive disorders, FGR, and neonatal birth weight [15,16] (Table 1).

**Table 1:** Similar studies evaluating the association of mid-trimester maternal serum homocysteine levels with pregnancy outcomes. tHcy = total serum homocysteine; FGR = fetal growth restriction; SGA = small for gestational age; PVD = placental vascular disease

Study	Year	Association of elevated tHcy with placenta-mediated disorders
Maru et al. [10]	2016	Significant association with hypertensive disorders, abruption, multiorgan dysfunction, and neonatal unit admissions
Cotter et al. [11]	2001	Significant association with hypertensive disorders in pregnancy
Laskowska et al [12]	2011	Significant association with FGR and hypertension
D’Anna et al. [14]	2004	No association with hypertensive disorders or FGR
Hietala et al. [15]	2001	No association with hypertensive disorders
Hogg et al. [16]	2001	No association with hypertensive disorders, FGR, or birth weight of babies
Dodds et al. [17]	2008	Significant association with hypertensive disorders, SGA, and pregnancy loss
Wang et al. [18]	2000	Significant association with hypertensive disorders, PVD, and FGR

Limitations of the study

The obvious shortcomings of this study, apart from the small sample size, are that it was conducted in a low-risk population, and no attempt was made to stratify women with a higher likelihood of developing pregnancy-related hypertensive disorders and FGR. The lack of adequate randomization and matching may help reinforce the concept of including serum homocysteine as an adjunct in women with a high risk of developing placental complications.

Literature analysis has emphasized that the homocysteine level in pregnancy is presumably related to geographical, ethnic, cultural, and social factors. Certain studies have also suggested alterations in the folate metabolism generated by MTHFR (methylenetetrahydrofolate reductase) polymorphism that impacts homocysteine metabolism. However, further introspections, including meta-analyses, are essential to define the cut-off and prognostic values of homocysteine in pregnancy, with an additional focus on determining the genetic factors of the disruptions in homocysteine metabolism.

## Conclusions

The study emphasizes the correlation of maternal serum homocysteine level with adverse pregnancy outcomes. Elevated homocysteine level was associated with the development of hypertensive disorders in the antenatal period, FGR, preterm birth (<37 weeks), and low Apgar score in neonates with excellent specificity. The encouraging outcomes with relevant clinical significance from the study only add to the increasing body of substantial evidence that can help promote the early identification and surveillance of placenta-mediated disturbances in pregnancy. Such a simple and affordable investigation has the potential to go a long way in the early diagnosis and management of these undesirable concerns in pregnancy during the antenatal period, especially in low-resource settings.

With ongoing explorations into the functionality of biomarkers in clinical research, the use of homocysteine as a diagnostic biomarker in pregnancy, particularly its specific etiological function in placental dysfunction, may require further relevant Mendelian randomization analyses.
